# Valid olfactory impairment tests can help identify mild cognitive impairment: an updated meta-analysis

**DOI:** 10.3389/fnagi.2024.1349196

**Published:** 2024-02-13

**Authors:** Chunyi Zhou, Chongming Yang, Yating Ai, Xueling Fang, Ailin Zhang, Yuncui Wang, Hui Hu

**Affiliations:** ^1^School of Nursing, Hubei University of Chinese Medicine, Wuhan, China; ^2^Research Support Center, College of Family, Home, and Social Sciences, Brigham Young University, Provo, UT, United States; ^3^Engineering Research Center of TCM Protection Technology and New Product Development for the Elderly Brain Health, Ministry of Education, Wuhan, China; ^4^Hubei Shizhen Laboratory, Wuhan, China

**Keywords:** mild cognitive impairment, olfactory function, cognitive function, meta-analysis, smell test

## Abstract

**Background:**

Olfactory testing is emerging as a potentially effective screening method for identifying mild cognitive impairment in the elderly population.

**Objective:**

Olfactory impairment is comorbid with mild cognitive impairment (MCI) in older adults but is not well-documented in subdomains of either olfactory or subtypes of cognitive impairments in older adults. This meta-analysis was aimed at synthesizing the differentiated relationships with updated studies.

**Methods:**

A systematic search was conducted in seven databases from their availability to April 2023. A total of 38 publications were included, including 3,828 MCI patients and 8,160 healthy older adults. Two investigators independently performed the literature review, quality assessment, and data extraction. The meta-analyses were conducted with Stata to estimate the average effects and causes of the heterogeneity.

**Results:**

Compared to normal adults, MCI patients had severe impairments in olfactory function and severe deficits in specific domains of odor identification and discrimination. Olfactory impairment was more severe in patients with amnestic mild cognitive impairment than in patients with non-amnestic MCI. Diverse test instruments of olfactory function caused large heterogeneity in effect sizes.

**Conclusion:**

Valid olfactory tests can be complementary tools for accurate screening of MCI in older adults.

## 1 Introduction

Cumulative evidence showed that olfactory impairment is comorbid with mild cognitive impairment and Alzheimer’s disease, with their common underlying neurodamages in the brain (Rahayel et al., 2012; Roalf et al., 2017; Dong et al., 2022; Pusswald et al., 2023). Olfactory impairment occurs earlier than visual impairment in MCI patients (Hagemeier et al., 2016) and predicts AD onset better than hearing and vision (Olofsson et al., 2020). As MCI usually harbingers AD, screening olfactory impairment has been recommended as a supplemental tool for identifying MCI (Jak et al., 2009); however, its efficacy remained uncertain in subdomains of olfactory and cognitive impairments.

Different aspects of olfactory impairment appeared to predict cognitive functions differentially. Olfactory impairment has been measured by detecting the minimum amount of odor (detection threshold), identifying a specific odor from a given list (identification), differentiating between odors (discrimination), and memorizing an odor and then identifying it (memory; Hedner et al., 2010). Odor detection threshold relies on the peripheral structural functions of the olfactory system and basic perceptual processing (Sohrabi et al., 2012), as opposed to odor identification and discrimination that involve higher brain centers and complex olfactory information processing systems (Stevenson and Boakes, 2003). Odor identification impairment was found to coincide with tau-mediated neuronal damage and occur before memory impairment and clinical symptoms in the course of AD (Bathini et al., 2019).

Subtypes of cognitive impairment include non-amnestic mild cognitive impairment (naMCI) and amnestic MCI (aMCI), which may be associated with olfactory dysfunction differentially. naMCI is more likely to progress to AD-unrelated dementia, such as frontotemporal dementia (FTD) or dementia with Lewy bodies (DLB; Devanand et al., 2015), and some studies suggest that both may be accompanied by severe olfactory dysfunction (Vyhnalek et al., 2015). However, this conclusion is controversial, as there are opposing studies showing that olfactory dysfunction in FTD and DLB patients is minimal or even absent (Luzzi et al., 2007). Most aMCI cases progress to AD dementia, caused by the degeneration of the internal olfactory cortex and hippocampus, which affects the individual’s ability to identify odors (Vyhnalek et al., 2015; Roberts et al., 2016). While aMCI patients usually exhibit more severe olfactory impairment than naMCI patients (Quarmley et al., 2017), others show similar or indistinguishable degrees of olfactory impairment between the aMCI and naMCI subtypes (Roalf et al., 2017) and odor identification deficits (Devanand et al., 2010; Vyhnalek et al., 2015). What complicates the association of olfactory impairments with MCI is that olfactory deficits in odor detection threshold, identification, discrimination, and memory can coexist in MCI patients (Yap et al., 2022). In addition, odor detection thresholds decline in the normal elderly population as well, though faster than discrimination and identification (Hummel et al., 2007). It remained uncertain how reliable it is to use olfactory impairment tests as supplemental tools for identifying MCI.

Earlier meta-analyses investigating olfactory function in MCI and AD patients have not differentiated domains of olfactory impairments and MCI subtypes. For instance, Roalf’s meta-analysis found that olfactory function in MCI patients is slightly worse than that in the normal population (Roalf et al., 2017), but only a few studies included MCI subtypes. In addition, there was no statistically significant impairment of odor memory in MCI patients due to the small number of included studies. Jung’s meta-analysis showed that MCI patients have significant deficits in odor identification compared to AD patients (Jung et al., 2019) but this did not extend to odor discrimination and detection thresholds. Other studies did not consider the heterogeneity of demographics, Mini-Mental State Examination (MMSE) scores, and olfactory test instruments (Wang et al., 2018; Yang et al., 2022). These different approaches to synthesizing previous findings have not rendered differentiated efficacies of olfactory impairment tests for identifying MCI.

This meta-analysis was to synthesize studies that might reveal the differentiated relationships between olfactory impairments and MCI, with a focus mainly on the following issues: (1) domains of olfactory impairment in MCI patients, (2) olfactory function in two subtypes of MCI patients, and (3) differences caused by test instruments of olfactory function, etc. This updated study differs from previous ones in that strict inclusion–exclusion criteria were applied in the literature search, only higher-quality and recent studies were included, and the whole research process adhered to the Preferred Reporting Items for Systematic Reviews and Meta-Analyses (PRISMA) 2020 statement that can be readily retrieved online (see Supplementary material).

## 2 Methods

### 2.1 Literature search strategy

The literature about olfactory function in MCI patients was searched in PubMed, Embase, the Cochrane Library, Web of Science, China Knowledge Network, Wanfang Data, and Vipul.com, within the time frame from their availability to April 2023. Using the Boolean logic for literature retrieval, the search strategy combined subject terms and free words without language restrictions as follows: “Cognitive Dysfunction” OR “Mild Cognitive Impairment” AND (“Smell” OR “Sense of Smell” OR “Olfaction” OR “Olfaction Disorders” OR “Olfaction Dysfunction” OR” Olfaction Impairment”) (see Supplementary material).

### 2.2 Inclusion and exclusion criteria

The inclusion and exclusion criteria for the studies complied with the requirements of the 2020 PRISMA. The inclusion criteria for this study were as follows: (a) The study subjects were MCI patients aged 50 years and older without co-morbidities or other neurodegenerative diseases. (b) There was a healthy population matched to the age of the MCI group as a control group. (c) The study subjects were MCI patients diagnosed by traditional methods. (d) The subjective olfactory function assessment was judged by the test subjects’ autonomous sniffing of odors. (e) The research design was a cohort or a case–control study.

Studies with the following characteristics were excluded from the meta-analysis: (a) the research reports omitted original effect sizes and the authors could not be contacted or provide them; (b) full reports could not be downloaded and accessed; (c) reports duplicated published data as determined by identical authors, study sites, participating institutions, details of olfactory tests, sample sizes, baseline situations, or study durations; (d) low-quality reports with a score of <7 on the Newcastle-Ottawa Scale (NOS) for assessing the quality of non-randomized studies.

**Figure 1:**
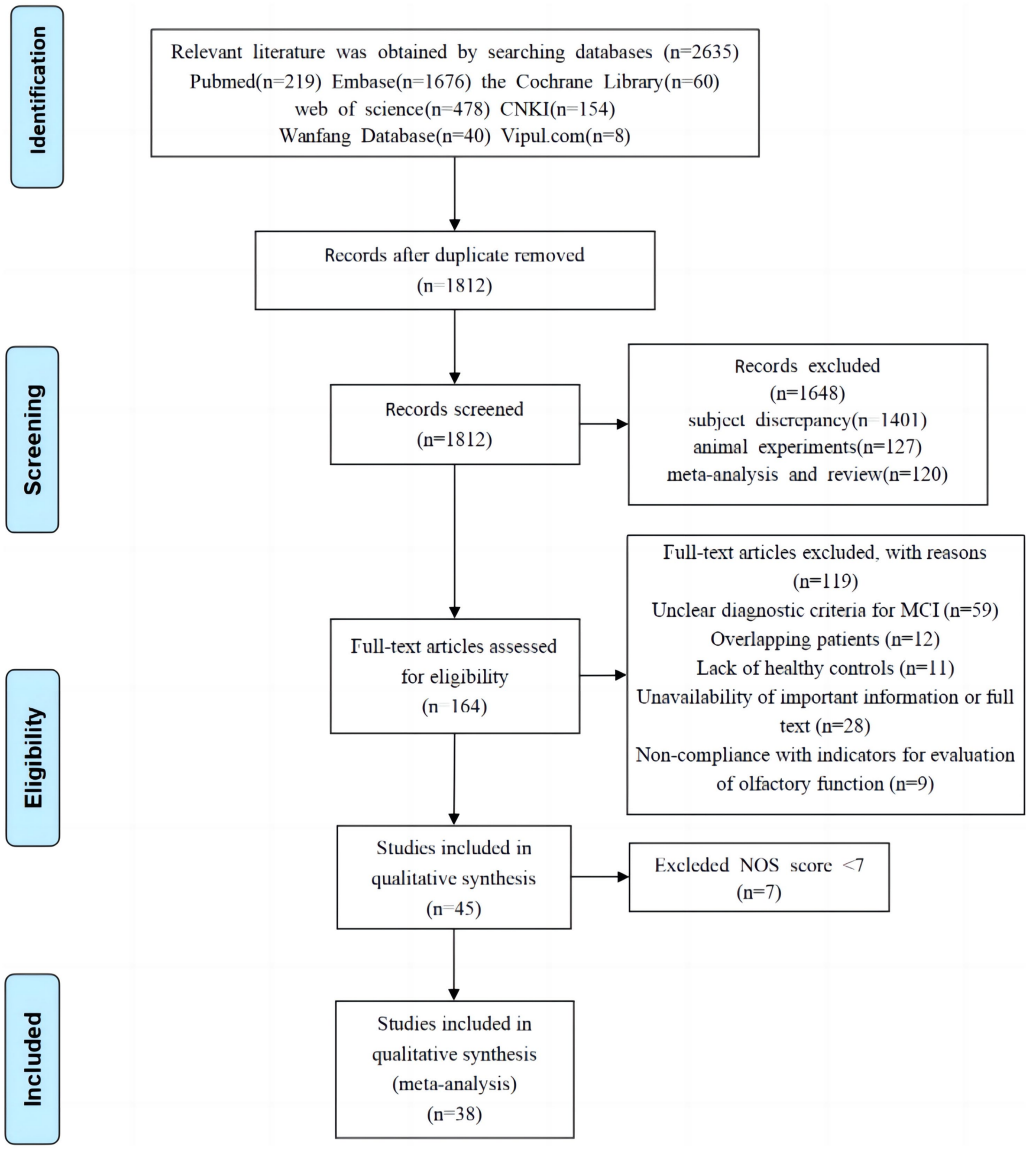
Flowchart of literature screening.

Figure 1 depicts the literature search and selection process for this study. The initial search yielded 2,628 pieces of pertinent reports in total. After meticulous screening, 30 case–control studies and 8 cohort studies were finally selected, including 12 from North America, 17 from Asia, and 9 from Europe. This meta-analysis comprised 3,828 MCI patients and 8,160 healthy controls.

### 2.3 Data extraction and quality evaluation

Data extraction and study quality evaluation were performed independently by two investigators of this study trained in evidence-based care according to the inclusion and exclusion criteria and cross-checked. A third investigator was requested to adjudicate any disagreement collaboratively. The extracted data included general information about the literature: title, year of publication, authors, country of study, type of study, sample size, age of participants in the control and MCI groups, olfactory function test instruments, and test scores of neuropsychological scales such as MMSE and MoCA. The quality of the included studies was evaluated using the NOS (Stang, 2010) and rated low (0–4 points), medium (5–6 points), or high (7–10 points). Only high-quality studies (scores ≥ 7) were included in this study. The literature was summarized and organized using Endnote X9 software, and data were extracted using Excel 2019. The fundamental characteristics and quality assessment of these studies are displayed in Table 1.

**Table 1:** Characteristics of the included studies.

Study	Country	Study type	Measures	MCI			HC			Neuropsychological scales	NOS score
				Sample size	Age	Olfactory function score	Sample size	Age	Olfactory function score		
Devanand et al. (2000)	USA	Case–control	UPSIT	90	66.70 ± 10.70	31.00 ± 7.40	45	64.00 ± 10.00	35.20 ± 3.90	MMSE	8
Wang et al. (2002)	China	Case–control	CC-SIT	28	71.90 ± 7.78	7.25 ± 1.41	30	73.84 ± 5.90	9.27 ± 1.26	MMSE	7
Peters et al. (2003)	Germany	Case–control	SSIT	8	72.50 ± 5.00	8.98 ± 2.83	8	73.90 ± 9.40	11.34 ± 2.48	MMSE	8
Tabert et al. (2005)	USA	Cohort	UPSIT	147	67.43 ± 9.85	31.22 ± 6.45	63	65.71 ± 9.38	34.86 ± 4.18	MMSE	7
Laakso et al. (2009)	Finland	Case–control	Homemade	72	73.00 ± 4.00	4.40 ± 2.48	486	71.00 ± 4.00	4.95 ± 2.42	MMSE	7
Yin et al. (2010)	China	Case–control	UPSIT	8	70.30 ± 11.00	25.40 ± 9.90	20	71.00 ± 10.00	30.80 ± 5.70	MMSE	7
Conti et al. (2013)	Italy	Cohort	CA-SIT	88	73.50 ± 6.78	11.68 ± 7.12	46	73.70 ± 7.30	14.25 ± 7.30	MMSE	8
Seligman et al. (2013)	USA	Case–control	SSIT	112	72.63 ± 8.19	10.10 ± 3.39	132	72.57 ± 9.52	12.55 ± 2.52	NA	7
Woodward et al. (2017)	USA	Cohort	UPSIT	110	74.05 ± 9.03	28.01 ± 7.97	194	72.29 ± 8.41	32.32 ± 5.47	MMSE	7
Huart et al. (2015)	Belgium	Case–control	Homemade	13	70.46 ± 5.97	23.90 ± 7.70	13	69.69 ± 8.35	27.00 ± 3.70	NA	8
Servello et al. (2015)	Italy	Case–control	SSIT	25	NA	9.15 ± 3.76	28	NA	10.35 ± 2.97	MMSE	8
Vasavada et al. (2015)	USA	Case–control	UPSIT	21	73.20 ± 9.00	24.20 ± 8.60	27	69.50 ± 10.40	34.00 ± 4.20	MMSE	7
Hagemeier et al. (2016)	USA	Case–control	UPSIT	19	NA	22.90 ± 8.60	19	NA	30.00 ± 6.70	MMSE	7
Liang et al. (2016)	China	Case–control	SSIT	345	73.00 ± 7.80	7.10 ± 2.30	1,437	69.40 ± 6.80	8.20 ± 2.00	MMSE	7
Kreisl et al. (2018)	USA	Cohort	UPSIT	46	68.80 ± 7.40	29.00 ± 6.40	25	67.90 ± 7.70	31.90 ± 5.80	MMSE	7
Quarmley et al. (2017)	USA	Case–control	SSIT	174	72.46 ± 8.57	9.94 ± 3.28	292	70.96 ± 8.74	12.43 ± 2.53	MoCA	8
Risacher et al. (2017)	USA	Case–control	UPSIT	5	75.70 ± 10.60	28.70 ± 7.00	19	68.50 ± 6.90	34.50 ± 2.30	MoCA	7
Tonacci et al. (2017)	Italy	Case–control	SSIT	85	74.60 ± 4.90	7.51 ± 3.72	41	73.50 ± 4.30	9.49 ± 3.87	MMSE	7
Umeda-Kameyama et al. (2017)	Japan	Case–control	OSIT-J	28	81.00 ± 6.00	5.00 ± 3.10	12	77.10 ± 6.40	7.30 ± 2.40	MMSE	7
Ward et al. (2017)	USA	Case–control	UPSIT	8	76.13 ± 6.29	21.63 ± 10.17	20	76.65 ± 6.48	33.60 ± 3.39	MOCA	7
Chen (2017)	China	Case–control	SSIT	63	67.80 ± 9.20	8.55 ± 3.02	57	65.20 ± 7.20	9.90 ± 3.00	MMSE	7
Woodward et al. (2018)	USA	Case–control	UPSIT	192	73.18 ± 9.05	26.98 ± 8.00	234	71.26 ± 8.05	32.19 ± 5.36	MMSE	8
Lu et al. (2019)	USA	Case–control	UPSIT	19	72.80 ± 9.40	25.58 ± 7.69	31	70.40 ± 10.00	33.42 ± 4.19	MMSE	7
Jiang (2019)	China	Case–control	UPSIT	24	65.00 ± 5.27	20.83 ± 5.56	30	62.13 ± 7.25	26.20 ± 3.75	MMSE, MoCA	7
Doorduijn et al. (2020)	Netherlands	Cohort	SSIT	22	69.80 ± 7.20	25.50 ± 1.40	40	62.50 ± 6.80	30.20 ± 1.10	MMSE	7
Kim et al. (2020)	Korea	Case–control	YSK OFT	26	74.96 ± 9.58	15.58 ± 6.36	104	69.30 ± 6.16	18.93 ± 5.07	MMSE	8
Iritani et al. (2021)	Japan	Case–control	OE	23	81.30 ± 8.10	3.55 ± 2.24	64	77.20 ± 5.90	6.14 ± 2.50	MMSE	7
Kjelvik et al. (2020)	Norway	Cohort	SSIT	17	74.40 ± 6.50	8.45 ± 3.10	28	67.40 ± 7.60	11.25 ± 3.02	MMSE	9
Chen et al. (2022)	China	Case–control	SSIT	118	67.90 ± 7.70	10.60 ± 2.30	50	64.50 ± 4.40	12.80 ± 2.00	MMSE	8
Suzuki et al. (2022)	Japan	Case–control	OE	26	77.40 ± 5.00	7.50 ± 2.20	12	73.10 ± 6.70	4.90 ± 2.00	MMSE	7
Yap et al. (2022)	Singapore	Cohort	Homemade	143	67.70 ± 5.90	5.30 ± 2.00	527	67.90 ± 5.20	5.70 ± 2.00	MMSE	7
Yi et al. (2022)	China	Case–control	SSIT	1,102	71.65 ± 5.04	7.80 ± 3.20	3,112	70.64 ± 4.46	9.25 ± 3.10	NA	8
Fukumoto et al. (2022)	Japan	Case–control	DESK	61	72.8 ± 10.6	14.7 ± 4.5	100	57.4 ± 11.7	18.4 ± 1.6	MMSE, MoCA	7
Tan et al. (2022)	China	Cohort	SSIT	157	68.8 ± 7.4	5.65 ± 2.44	447	67.9 ± 7.7	6.35 ± 2.23	MMSE, MoCA	8
Delgado-Lima et al. (2023)	Spain	Case–control	SSIT	55	77.5 ± 6.47	9.67 ± 2.44	46	72.6 ± 5.56	12.7 ± 2.05	MoCA	9
Liang et al. (2023)	China	Case–control	CSIT	85	76.0 ± 7.8	12 ± 3.02	135	71.7 ± 8.1	14.67 ± 0.75	MMSE, MoCA	8
Guo et al. (2023)	China	Case–control	SS-16	75	70.5 ± 5.2	10.7 ± 2.1	50	70.5 ± 5.0	11.7 ± 1.8	MMSE, MoCA	7
Mi et al. (2023)	China	Case–control	CSIT	188	62.45 ± 7.15	13.06 ± 2.05	136	60.43 ± 7.61	14.6 ± 1.57	MMSE, MoCA	8

### 2.4 Olfactory function test instruments used in the included studies

The *University of Pennsylvania Odor Identification Test (UPSIT)* is a forced-choice odor identification assessment in which each subject is sequentially exposed to 40 odors and scores 1 point for each correctly identified odor (Doty et al., 1984).

The *Sniffin’ Sticks Identification Test (SSIT)* was developed in Germany to measure detection threshold, discrimination, and identification function (Hummel et al., 1997). Subjects were presented with 16 felt-tipped pens containing common household odors, requested to freely identify each with a verbal description, and scored one point for each correct identification.

The *Japanese odor stick identification test (OSIT-J)* is an identification tool to identify odors familiar to Japanese patients (Shino et al., 2006). Subjects were required to sniff out a target odor from four samples and choose “detectable but unrecognizable” or “no odor detected” (no score). A correct identification was scored 1 point.

The *Open-Essence (OE) test* is a similar card-based odor identification tool with 12 odors, designed to overcome the inconvenience of odor sample storage (Okutani et al., 2013).

The *culturally adapted version of the odor identification test (CA-SIT)* is an Italian culture-adapted version of the UPSIT, with six odors removed from the original, which can be easily misidentified by Italians (Doty et al., 1996) and scoring similar to the UPSIT.

The *cross-cultural version of the olfactory identification test (CC-SIT)* is a cross-cultural version of the UPSIT (Doty et al., 1996) that consists of 12 odors familiar to US, Chinese, French, and Japanese patients. One point was scored for each correct identification, up to a total of 12.

The *YSK Olfactory Function Test* is a Korean olfactory threshold test used for early screening for dementia in older adults (Kim et al., 2021). The tool consists of a series of kits, such as odorless distilled water as a blank stimulus and 10-step concentrations (0%–16%) of rose-scented 2-phenylethanol. Scores range from 1 to 7, with lower scores indicating higher olfactory thresholds.

The *DEmentia Screening Kit (DESK)* is an odor identification test tool developed for Japanese patients with dementia or AD (Fukumoto et al., 2022). The kit includes 10 odorants in 2 concentrations (weak/strong), for a total of 20 combinations. Two different concentrations of odors were tested separately with a paper cup each time and a 5-min interval between. Patients were requested to choose an answer from six alternatives to indicate whether they could identify an odor, and they scored 1 point for each correct odor identification.

The *Chinese Smell Identification Test (CSIT)* is an odor identification test developed by the Institute of Psychology of the Chinese Academy of Sciences in 2019 which contains 40 or 16 odors familiar to Chinese patients (Feng et al., 2019).

In addition, researchers of the three studies created “homemade tests” for participants to identify, discriminate, or detect odors. These could be as simple as small containers (e.g., jars or vials) filled with different scents (e.g., essential oils or spices) for the participants to smell and identify.

### 2.5 Data analysis

Data from the included studies were meta-analyzed using the statistical software Stata (v14). The group mean differences in olfactory function scores were converted to standardized mean differences (*SMDs*), which are also referred to as Cohen’s *d* to render differences commensurate across the studies (small ≤ 0.2, medium = 0.05, and large ≥ 0.8; Cohen, 1977). Medians and quartiles were converted to means and standard deviations using the methods of Luo et al. (2018) and Wan et al. (2014). Random effect models (REMs) were employed to estimate the average effect sizes and heterogeneity (*I^2^*) between studies (unavailable *for a* single study), whose sources were further explored through subgroup analyses and meta-regressions. *I^2^* ≥ 50% and *p* ≤ 0.05 indicate high heterogeneity between studies and the necessity of REM. Sensitivity analysis was also conducted to detect the influence of individual studies on the average effect size. Egger’s test was used to examine the presence of publication bias, with a *p*-value of ≤0.05 indicating the presence.

## 3 Results

### 3.1 Literature screening process and overall effect size

The initial research yielded 2,635 pieces of pertinent literature in total. After meticulous screening, 30 case–control studies and 8 cohort studies, including 12 from North America, 17 from Asia, and 9 from Europe, were finally included. The final meta-analysis comprised 3,828 MCI patients and 8,160 healthy controls. Table 2 displays the fundamental characteristics as well as the quality assessment of the selected studies. The overall effect size obtained from a random effect model was *SMD* = −0.78, 95% CI: −0.89~−0.66, *I^2^* = 81.3%.

**Table 2:** Meta-regression results.

Results	Region	Age	Education	Design	MMSE	MoCA
Regression coefficient	−0.03	−0.06	−0.02	−0.09	−0.03	−0.08
*95%CI*	−0.29~0.24	−0.35~0.24	−0.24~0.27	−0.52~0.33	−0.18~0.13	−0.14~−0.02
*P*	0.846	0.702	0.880	0.662	0.727	0.017

### 3.2 SMD by regions, participants’ age, and education

**Figure 2:**
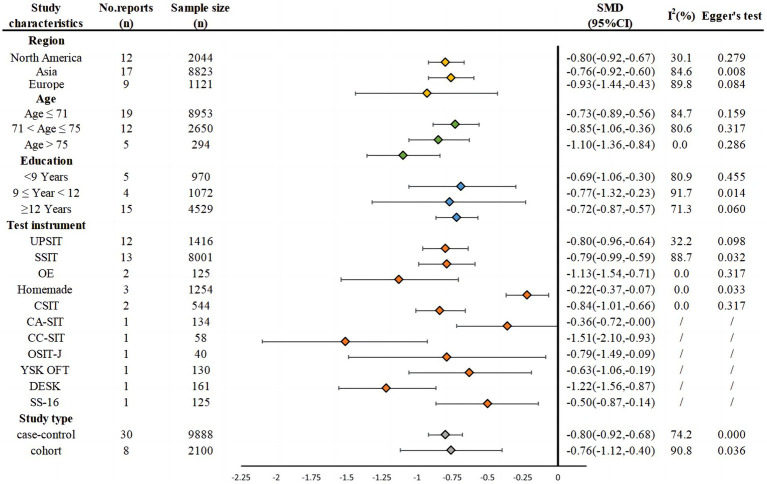
Basic characteristics of the studies and SMD by different moderating variables.

Figure 2 presents the basic characteristics of the 38 included studies and SMD by certain potential moderators. First, *SMD* was −0.80 (95% CI: −0.92~−0.67, *I^2^* = 30.1%) for 12 studies in North America, −0.76 (95% CI: −0.92~−0.60, *I^2^* = 84.6%) for 17 studies in Asia, and −0.93 (95% CI, −1.44~−0.43, I2 = 89.8%) for 9 studies in Europe.

Second, 3 age groups had *SMD* = −0.74 (95% CI: −0.93~−0.55, *I^2^* = 89.4%) in 15 studies with participants’ mean age ≤ 71 years, *SMD* = −0.78 (95% CI: −0.98~−0.58, *I^2^* = 75.4%) in 11 studies with participants’ mean age between 71 and 75 years, and *SMD* = −0.96 (95% CI: −1.29~−0.64, *I^2^* = 0%) in 4 studies with participants’ mean age greater than 75 years.

Third, 3 education groups showed *SMD* = −0.82 (95% CI: −1.33~−0.31; *I^2^* = 80.0%) in 4 studies whose participants had less than 9 years of education; *SMD* = −0.57 (95% CI: −1.46~−0.57; *I^2^* = 83.9%) in 3 studies whose participants had between 9 and 12 years of education; and *SMD* = −0.72 (95% CI: −0.87~−0.57; *I^2^* = 71.3%) in 15 studies whose participants had more than 12 years of education.

### 3.3 SMD by test instruments

The effect sizes also varied by test instruments used in the included studies, with *SMD* = −0.80 (95% CI: −0.96~−0.64, *I^2^* = 32.2%) by UPSIT; *SMD* = −0.79 (95% CI: −0.99~−0.59, *I^2^* = 88.7%) by SSIT; *SMD* = −1.13 (95% CI: −0.96~−0.64, I2 = 32.2%) by OE; *SMD* = −0.22 (95% CI: −0.37~−0.07) by homemade instruments; *SMD* = −0.84 (95% CI: −1.01~−0.66) by CSIT; *SMD* = −0.36 (95% CI: −0.72~−0.00) by CA-SIT, *SMD* = −1.51 (95% CI: −2.10~−0.93) by CC-SIT, *SMD* = −0.79 (95% CI: −1.49~−0.09) by OSIT-J, *SMD* = −0.63 (95% CI: −1.06~−0.19) by YSK OFT, *SMD* = −1.22 (95%CI: −1.56~−0.87) by DESK, and *SMD* = −0.50 (95% CI: −0.87~−0.14) by SS-16.

### 3.4 SMD by studies designs

Two types of research design had *SMD* = −0.80 (95% CI: −0.92~−0.68; *I^2^* = 74.2%) in the 30 case–control studies and *SMD* = −0.76 (95% CI: −1.12~−0.40; *I^2^* = 90.8%) in the 8 cohort studies.

### 3.5 SMD by olfactory function domains in 38 studies

**Figure 3:**
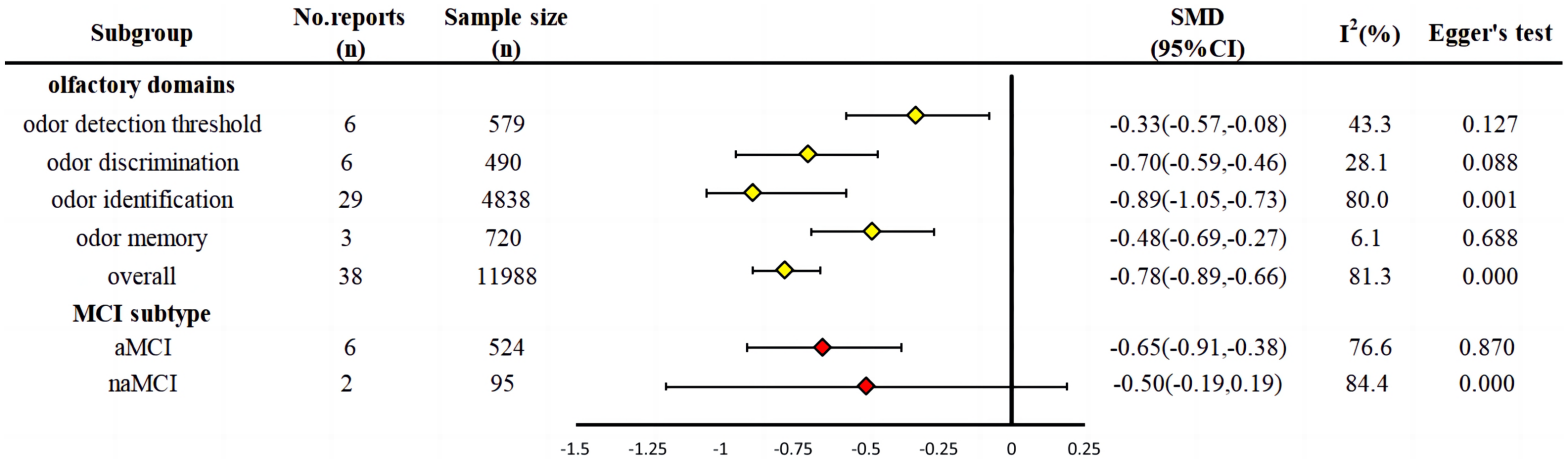
Subgroup analysis.

As shown in Figure 3, MCI patients were lower than the healthy controls in odor detection thresholds (*SMD* = −0.33, *95% CI:* −0.57~0.08, *p* < 0.001), memory (*SMD* = −0.48. *95% CI:* −0.69~−0.27, *p* < 0.001), discrimination (*SMD* = −0.70, *95% CI*: −0.59~−0.46, *p* < 0.001), and identification (*SMD* = −0.89, *95% CI*: −1.05~−0.73, *p* < 0.001) in ascending order of the effect size.

### 3.6 SMD by subtypes of MCI in six studies

The degrees of olfactory impairment were more severe in the aMCI group (*SMD* = −0.65, 95% CI: −0.91~−0.38, *p* = 0.001) than in the naMCI group (*SMD* = −0.50, 95% CI: −0.19~0.19, *p* = 0.155), where the difference in the naMCI group was not statistically significant from zero, as shown in Figure 3. Six studies reported patients with aMCI, with a total sample size of 524 cases (*I^2^* = 76.6%, *p* = 0.001). Two studies reported patients with naMCI with a total sample size of 95 cases (*I^2^* = 84.4%, *p* = 0.005). The heterogeneity between the two groups was *I^2^* > 50%, as indicated by a random effect model.

### 3.7 Heterogeneity by demographics and MCI tests

As shown in Table 2, meta-regression was performed on the effect-coded region (Daly et al., 2016), age, education levels, study design, and cognitive test scores (MMSE and MoCA). The results showed that olfactory impairment varied only with MoCA scores when pitted against one another in the model.

### 3.8 Sensitivity analysis and publication bias

Publication bias was evaluated by Egger’s test (*p* = 0.372), which suggested that there was no significant evidence of publication bias. After excluding studies one by one, the effect size and the 95% CIs showed robustness in all results (see Supplementary material).

## 4 Discussion

The current meta-analysis of 38 high-quality studies revealed that MCI patients had much lower overall olfactory function than the healthy participants in terms of the large effect size. The overall difference also varied across domains of olfactory function, test instruments of olfactory function, subtypes of MCI (naMCI and aMCI), and cognitive tests of MCI.

The effect size in the overall olfactory function was close to the large effect found in the early meta-analysis that compared MCI patients with normal adults (Roalf et al., 2017), but much less than those in studies that compared AD patients (Rahayel et al., 2012; Vyhnalek et al., 2015; Roberts et al., 2016; Kotecha et al., 2018; Jung et al., 2019). This was expected, as the included studies also compared MCI patients with normal adults.

Specific domains of olfactory function in this meta-analysis showed that MCI patients had the most drastic lower function in odor identification and discrimination than normal adults, in terms of the effect size (Cohen, 1977). These large effects imply that these tests may be used exclusively or conjointly with other MoCA to identify MCI in clinical settings. In contrast, the smaller effects in odor detection thresholds and odor memory imply that these tests cannot differentiate MCI patients from normal aging adults (Hummel et al., 2007), and thus may be recommended to the general public to guard against any further deterioration and symptoms of MCI due to neurobiological changes.

Test instruments of olfactory function appeared to contribute largely to the overall olfactory function differences between the MCI and normal individuals, with the effects ranging from the smallest by homemade instruments (*SMD* = −0.22) to the largest by CC-SIT (*SMD* = 1.51). Homemade odor samples could have been familiarized and sensitized to the participants in the three studies, so that the odors in these studies could also be detected, identified, or discriminated against easily by MCI patients, resulting in small effect sizes. Small effect sizes suggest that the odor test instruments were not as sensitive and efficacious as those that yielded larger effects. Therefore, certain odor test instruments may be improved for better validity/efficacy.

The aMCI subtype patients exhibited more pronounced deficits in odor identification and discrimination than those with naMCI (*SMD* = −0.65>0.50), which suggests that olfactory impairment is associated with aMCI (Vyhnalek et al., 2015; Roberts et al., 2016). However, as the effect size of the two studies that involved naMCI patients was non-significant due to small samples and few studies, the differential predictive powers of the two subtypes of MCI need to be ascertained with further studies, especially prospective longitudinal ones.

The meta-regression suggested that the effect sizes were similar across regions and age and education groups when controlled for MCI measures and study designs. This suggests that olfactory function between MCI and normal adults was similar across regions, age groups, and education groups. This might also suggest that the underlying mechanism for the association of olfactory dysfunction with MCI can be universally bio-neurological and that olfactory impairment could be a reliable biomarker and predictor for cognitive decline (Deary et al., 2009; Windon et al., 2020).

MoCA outweighed MMSE in moderating the overall effect size. This may be because the MoCA scale assesses a broader range of cognitive domains, including executive function, visuospatial ability, and language, and is more sensitive in detecting mild cognitive impairment (MCI) and early dementia than the MMSE. MoCA also has fewer ceiling effects than the MMSE by including tasks that are challenging even for high-functioning individuals (Jia et al., 2021). Thus, the MoCA scale appeared to be more sensitive for screening early cognitive decline (Shao et al., 2021).

Despite the rigorous selection of high-quality studies and stringent inclusion criteria applied in this research, it is unfortunate that a considerable degree of heterogeneity persists. This may be due to the inclusion of a wide array of olfactory tests, each assessing different domains of olfactory function. Variability may also arise from the application of identical olfactory tests across diverse populations and countries, leading to potential discrepancies in outcomes. The Sniffin’ Sticks Identification Test (SSIT) in particular demonstrated the greatest heterogeneity, which can be attributed to the multiplicity of testing methodologies employed in various countries, each with its unique approach. Moreover, within the subgroup analysis of olfactory domains, olfactory identification tests revealed the most pronounced heterogeneity. This is likely a consequence of the global emphasis on olfactory identification abilities in the majority of olfactory assessment tools, contributing to a broad spectrum of tools and types and, thus, heightened heterogeneity.

## 5 Limitations and future directions

Although this study provides valuable insights into the relationship between olfactory impairment and MCI, several limitations must be considered. First, the high heterogeneity in this study may have been influenced by clinical factors, such as smoking, alcohol consumption, genetic factors, and COVID-19 infection status, which were not controlled for in the included studies. Second, the limited number of original studies on olfactory impairment in different subtypes of MCI patients means that they did not permit explorations of more complicated interactions between olfactory subdomains, subtypes of MCI, and other factors. Future studies may adopt prospective longitudinal designs, improve olfactory function tests, and probe into the neurological causes of the association between olfactory and MCI.

## 6 Conclusion

Olfactory impairment accompanies MCI, but the magnitude of the association depends on the measured domains of olfactory function, test instruments to measure both olfactory function and MCI, and subtypes of MCI. Severe deficits in odor identification and discrimination are more associated with MCI in aMCI patients. Valid odor identification and discrimination tests are recommended to complement MoCA and improve screening accuracy.

## Data availability statement

The original contributions presented in the study are included in the article/Supplementary material, further inquiries can be directed to the corresponding authors.

## Author contributions

CZ: Data curation, Writing – original draft. CY: Formal analysis, Methodology, Writing – review & editing. YA: Data curation, Writing – review & editing. XF: Data curation, Formal analysis, Writing – review & editing. AZ: Investigation, Visualization, Writing – review & editing. YW: Funding acquisition, Methodology, Supervision, Writing – review & editing. HH: Funding acquisition, Resources, Writing – review & editing.
